# The Aesthetic Turn in Mental Health: Reflections on an Explorative Study into Practices in the Arts Therapies

**DOI:** 10.3390/bs8040041

**Published:** 2018-04-23

**Authors:** Rosemarie Samaritter

**Affiliations:** Master of Arts Therapies Programme, Codarts University of the Arts, Kruisplein 26, 3012 CC Rotterdam, The Netherlands; rasamaritter@codarts.nl

**Keywords:** arts therapies, dance therapy, music therapy, aesthetics, mental health

## Abstract

The paper will draw on materials from arts therapies literature and comments from expert panels to discuss some specific characteristics of the arts therapies and to investigate the role of aesthetic engagement for resilience and mental well-being. The arts increasingly find their way as interventions in mental health domains. However, explorations into the specific mechanisms that underpin the therapeutic effect of arts-based activities are still scarce. Qualitative data were collected from a thematic literature review and experts’ comments on meaningful working procedures in arts therapies. Analysis of multiple data sources revealed core themes and core procedures that occur across arts therapy modalities. This paper presents a practice informed model of arts-based methods in mental health that may serve as a conceptual frame of reference for arts therapists and as study material on the applicability of arts therapy interventions for specific mental health settings.

## 1. Introduction

The arts are present in many public health domains. The beneficial effect of participation in cultural activities is more and more considered in the politics of health care [1,2]. In the Netherlands, the term ‘cultural intervention’ has been coined for the work of performers and art makers in care institutions, which is meant to promote participation in cultural or artistic activities for specific communities or groups with special needs. The current rediscovery of the arts as a socio-cultural intervention for the support of wellbeing and the inclusion of vulnerable or isolated social groups has also an impact on the practice of the traditional arts therapies (ATs), which cover the modalities dance, music, drama and visual arts. 

The ATs are integrated in a broad range of mental health circuits. They occur in psychiatric and psychotherapeutic settings, psycho-education, special needs education, psycho-social care and prevention. Relevant evidence for the efficacy of these interventions is of great importance in view of the economic and political development of the mental health field [3]. Identification of elements or factors that contribute to the effects of arts-based therapies is necessary to explore and discuss their specific contribution to wellbeing and mental health and their usefulness with specific populations [4]. However, it is a challenging task to translate the complexity of arts-based activities into linear conventional research structures [5]. There are good reasons to argue that the exploration of working mechanisms in the ATs needs to follow the experiential nature of the subject [6]. In this perspective, the experiences of practitioners as well as participants in ATs may hold important information on their tacit knowledge about working factors and procedures in ATs. 

This paper had its origin in a qualitative study that aimed to collect implicit models on the experiential nature of arts-based interventions among faculty and students of an ATs master training in the Netherlands. It will summarise the literature and the qualitative analysis and will offer a conceptual model of core themes and core procedures across the ATs modalities. 

## 2. Art and Therapy

**Figure 1:**
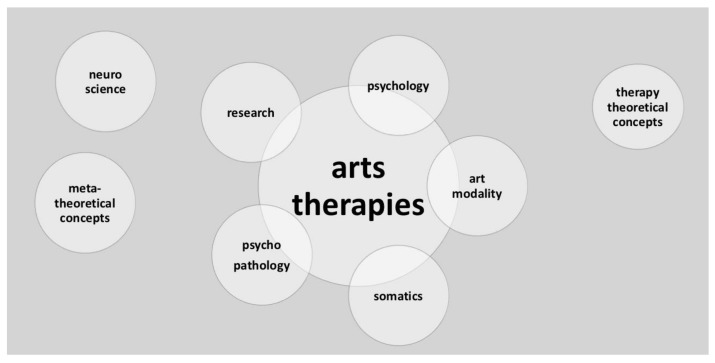
Multi-disciplinary position of the arts therapies.

Throughout history arts have been applied in the context of healing and recovery (Sachs, 1984) [7] Music, Dance, Art and Drama have been applied in therapeutic settings since the late 19th century, early 20th century and developed into professional mental health applications after the second world war [8]. Situated in psychiatric hospitals, ATs in the early years were offered as day activities to prevent deprivation and keep patients busy throughout the day. It showed that working with the arts also had an impact on the mental, somatic and psychological condition of the patients involved [6]. Around the first world war, psychiatrists and practitioners discovered that emotional balancing occurred after artistic activities. Patients seemed to be able to express their inner worlds in the arts in a way they had not been able to express through words [9] and articulation of experiential content led to catharsis from psychological distress [10]. When the use of arts in psychiatry developed into more systematic therapeutic approaches, practitioners also sought for theoretical models that could frame the methodological application and observed outcome of arts-based interventions. Throughout the second half of the 20th century the ATs developed a theoretical framework for the clinical practices as well as methodologies for specific patient populations. ATs presented a theoretical conceptualisation based on psychology and psychotherapeutic schools [11,12] and developed towards specific therapeutic applications for a diversity of populations and problems [13]. Being part of mainstream mental health care nowadays, the ATs contribute to diagnostics and treatment of psychological and medical conditions. Arts therapists need to be experts in the psycho-social as well as somatic aspects of their work, they need to develop skills in their art modality and knowledge about the therapeutic potentials of their art form in order to responsively support a broad variety of populations towards recovery or stabilisation. Figure 1 shows the multidisciplinary professional reality of arts therapists, which in itself forms a complex interplay. Recently arts therapists face the challenge to show evidence and effectiveness of their work. Interventions need to be described and manualised to design suitable research projects. 

## 3. Art as Experience

Experiences in the arts, such as playing music in an ensemble, choreographing a group of dancers or sharing the intentional space with a dance partner provide an experiential knowledge about the implicit processes of attunement between participants. They cover a broad variety of actions, such as orienting towards a partner or the artistic material, engaging in shared play or synchronizing in shared activities. These processes may be structured by composition or they may be open like in improvisational formats. In both cases the artistic actions are meant to bring about an aesthetic experience in artists as well as in the audience. Aesthetic here is not understood as a category of beauty or a specific artistic normativity. Although the concept of beauty may apply, aesthetic here is considered a specific experiential constellation that speaks to the maker as well as the perceiver as something that feels fitting well with the personal sensory-expressive state or that feels as meant to be [14]. This aesthetic appeal usually appears within an interaction of the individual with the arts materials (colours, movements, sound); and/or as an interactional quality of addressing or being addressed within the intersubjective encounter between players, makers and audience. The aesthetic appeal bears the potential of evoking new experiences in all participants, irrespective of whether they take an active role as a maker or performer or a passive role as the audience. 

The participants bring their previous experiences, preferences, sensory capacities and expectations to this aesthetic encounter, which will in turn have an impact on these aspects and will offer the opportunity for the participant to integrate new and old and come out of the aesthetic experiences with a change of intra- and intersubjective constellations [14,15]. The novelty that is intrinsic to each aesthetic experience, as it happens just in that present moment for the participant(s), thus allows for learning [16]. 

According to Brandstätter, arts can bring about an aesthetic touch [17]. Within the artistic activity or while watching a performance the actor as well as the onlooker feels part of the situation. One comes to a performance or a gallery with the willingness to open the senses to the makings of the artist, with a willingness to enter an aesthetic experience. The same is true for looking at a piece of visual art or listening to music. While looking or listening one feels a response to the perceived. The inward response (this is beautiful; this is meant for me; this moves me) occurs together with the feeling and knowing that this perception is directly related to the perceived. It is in this relation of inward sensing and outward appreciation that we can discern the beautiful form the ugly, the syntonic from the dystonic. It is not an objective standard that identifies a situation as being aesthetic or that turns a work into art work but it is an experiential engagement between the maker and the material, between the performer and the audience or the piece of art work and the onlooker, that contributes to an aesthetic unfolding of the situation [18]. It is an essentially experiential orientation that is characterised by the aesthetic engagement rather than by the mere perception of an art work as such. The aesthetic unfolding thus happens when an individual is simultaneously engaged in inward deepening of lived experiences and outward appreciation of and meaning assignment to a phenomenon or event [19]. As will be discussed later, this simultaneity is also crucial for the ATs, in that aesthetic experiences are not a matter of a particular series of acts or activities that can be performed subsequently but rather they are complex events in which inward experience and outward appreciation are defining each other and evolve within the dynamics of the enactively engaged therapist-patient-system [20].

## 4. Aesthetic Engagement in the Arts Therapies: An Explorative Study

The current project was conducted as an explorative study among faculty, students and alumni of a Dutch ATs master programme. The project was approved by the institute’s research board. The aim of the project was to collect and describe ATs experts’ models on the therapeutic mechanisms of arts-based interventions. Experts’ descriptions of meaningful experiences in their ATs professional practice were considered suitable data sources to capture the implicit models on ATs processes. The study was conducted in a modified grounded theory approach [21] with mixed data sources. 

### 4.1. Data Collection and Analysis

Initial data were collected by means of a literature study, expert circles with music therapy (MT and dance movement therapy (DMT) faculty members and a survey on key experiences in the ATs under students, alumni and faculty. Sources from ATs literature and Scopus searches on ‘arts therapies’ and ‘aesthetic experience/engagement’ were analysed for presented themes and practices, with a frame of five core themes that originally had been developed from the author’s DMT background [22]. The narrative summary of obtained themes and practices was then presented and discussed in a faculty expert panel (FP). A Delphi method [23] was applied to explore if the themes retrieved from the literature covered faculty’s perspectives on the therapeutic use of arts informed practices. In a second step an online expert survey on meaningful experiences in ATs (ES) was send out to broader faculty, students and alumni. This research step was meant to critically review the themes and procedures that were identified during the previous steps. The survey consisted of nine questions. Three demographic items inventoried background (student/professional/MT/DMT) and experience (years of experience; ATs target groups) of the respondent. Six items inventoried meaningful experiences from personal as well as professional participation in ATs activities. Content analysis of these materials was used to critically review and annotate the themes that had been identified during the previous steps. In a supplementary step the obtained core themes and procedures were explored for their feasibility as observational categories for the analysis of ATs practices as taken from video vignettes (AV) available from the programme’s classes and workshops. Data analysis followed a modified grounded theory approach with the three data sources (FP, ES, AV). The qualitative analyses of all sources were summarised in overarching themes and structures.

Throughout all research steps the institute’s ethical procedures were followed. Informed consent was given by participants in the faculty expert circle and for the use of video-materials. The online survey was distributed among faculty, students and alumni by email, with a link to the survey. Addressees were free to decide on their participation anonymously. Completion of the survey was estimated to take fifteen to thirty minutes; no incentives were offered. Participants could leave the questionnaire at any time, questions could be skipped, some allowed for multiple answers. To ensure anonymity, no identifying information was collected and return function was built without electronic tracking. 

### 4.2. Participants

Participants in the expert circle were six faculty members of the ATs programme, three MT, two DMT, one other discipline. All with extensive experience in ATs education and/or ATs clinical settings. Response rate in the online survey was with 16% relatively low. However, 58% of the addressees were alumni. It could not be verified whether addresses were still up to date in all cases and thus invitations may not have reached everyone, which in turn may have affected the return rate. Participants in the online survey were nineteen ATs experts, students MT (10,53%) and DMT (10.53%), certified therapists MT (10.53%)/DMT (47.37%), practising therapists MT (5.26%)/DMT (47.37%), tutor in MT programme (15.79%), tutor in DMT programme (26.32%). For ethical reasons, no specific demographic data were collected about gender, age or ethnicity. With the researcher being involved in the curriculum and the closer network of the training programme this would have allowed for identification of individuals, which might have caused addressees feeling compelled to participate.

### 4.3. Results

The data from the FP and ES were analysed as two independent sources. The analysis of the contributions from the FP resulted in the (re-)formulation and annotation of five ATs core themes. The content analysis of ES on meaningful experiences in ATs did not yield any additional themes. The focus here seemed to be more on procedures in ATs. The qualitative analysis of these two data sources were then summarized into two global domains, (i) *core themes* that are present or addressed in ATs; (ii) *core procedures* and practices that are used in ATs. A third domain emerged on the background of the analysis of themes and procedures, which was indicated as *leadership styles* in ATs. The latter was not further analysed in the course of the current project. 

*(i) Core Themes in ATs*

From the thematic analyses of ATs literature five overarching themes were formulated. Following the Delphi method these themes were discussed in the FP, the discussion was summarized and then presented to the FP for additional reflection and comments. After further annotation, FP found consensus on the five themes. FP indicated that in music and dance these five aspects are usually present simultaneously. The following paragraphs will present the five core themes with some specific examples from the FP and references to examples from the literature. 

(a) Embodied presence: arts support and require embodied presence. For DMT this occurs in the form of kinaesthetic listening [24] and a readiness to move. For MT this occurs in the form of tonal or musical presence [25] and a readiness to play. For both disciplines experts mentioned the specific aspect of (multi-)sensory processing and acting [26].

(b) Somato-sensory engagement: arts support anatomical, visceral and neuropsychological functioning. DMT and MT make an appeal on somatic resources, both have a vitalising effect. Also, for both disciplines FP and ES mentioned the effects of engagement in dance or music for neurological synchronisation and (re-) patterning [27,28]. 

(c) Emotional engagement: arts support articulation and expression of emotional content. Music therapists mentioned articulation and expression of inner feelings through different music instruments, or different musical rhythms or colours. Dance movement therapists pointed to articulation and expression of inner feelings in specific movement qualities and narrative expression through movement sequences. 

(d) Nonverbal communication: arts support sensitivity to non-verbal communication between participants. During FP and ES dance therapists and music therapists mentioned the important contribution of arts-based interventions to non-verbal attunement and communication. Also, enactive empathy was mentioned as a core aspect of both modalities [20,29]. 

(e) Intercultural involvement: Arts support social and cultural interaction. Experts mentioned the possibility to develop a sense of social relatedness through participation in group dance or music. The shared playful experiences were mentioned to contribute to empathetic sense making between participants. The non-verbal or pre-verbal character of aesthetics was considered to support diversity and cross-cultural interaction [30].

(*ii) Core Procedures in ATs*

Methods in ATs are diverse, multi-layered and heterogeneous due to the use of the creative processes. However, the literature describes for all ATs procedures to develop the patient’s potential for sensing, articulating and expressing, for being an agent and a maker [30,31]. FP emphasized the importance of the use of procedures such as improvisation, playing together, composing or choreographing. In addition, the results from the ES revealed more specific information on ATs procedures. The described procedures were analysed for the composing actions, which the researcher then related to basic actions that occur across all art modalities. 

Artists invite and address their audience. They engage themselves and others in the arts modality. They play, explore, select, compose and replicate their materials and activities towards a performance into which an audience is invited to engage, explore and play within the performed. Throughout the process of art making and presenting re-occurring experiential and procedural loops can be identified that cover the activities between initial improvisation, setting material, presenting and performing the material. Table 1 gives an overview on these arts related activities and procedures and their application in ATs. 

For the context of this paper these procedures may be considered as arts-informed therapeutic procedures for the ATs to differentiate from for example psychological informed procedures such as psycho-analytical, behavioural or mentalization-based models. In FP and ES, the described procedures were mentioned to be situated within an intersubjective engagement between patient and therapist. From the VA a selection of video vignettes was prepared to illustrate the obtained themes and procedures.

## 5. Discussion

The results on the core themes suggest that aesthetic engagement in the ATs is to be understood as an experiential quality that captures the simultaneity of sensory-expressive-relational-socio-cultural involvement that is characteristic for arts practices. The therapeutic working alliance in ATs is embedded in this experiential complexity. During the (co-)creation within the arts modality aesthetic engagement emerges, deepens or fluctuates [32]. 

In a healthy condition, humans do have the capacity for aesthetic engagement, for being in the present moment and experiencing animated affects and expressivity [33], for creativity to handle and overcome challenging life events. However, burdening life experiences or psychological conditions may have an impact on the capacity for creative and aesthetic engagement [34]. This may lead to an imbalance of the adaptive capacities in the present moment and to anaesthetisation from vitality. 

Examples in the ES revealed that for specific pathologies, the emphasis within the aesthetic activities may differ. Working from the core themes allows the therapist to focus on a patient’s pathological issues, while taking into account the aesthetic deepening that occurs through the interplay of these themes. The following paragraph will discuss some examples of pathological issues in relation to the ATs core themes and will give some references to research on the effectiveness of ATs interventions for the respective issue. 

Patients with personality disorders, dissociative disorders, severe depression or trauma often describe a blocked perception of bodily feelings/perception and a disturbed sense of self. A therapeutic approach that helps to develop embodied presence would therefore be a first step to develop some sense of self, that then could serve as a point of reference throughout the therapy process [35]. Patients with neurological disorders may suffer a distorted movement organisation, like in Parkinson’s Disease. Somato-sensory (re-)patterning has been described as a basic intervention to allow for patients to develop a regulated sensory-motor organisation and reconnect to bodily organisation [36]. Patients with severe mood disorder may suffer from blocked feelings. Arts-based interventions allow for (re-)connection with hidden feelings and for their articulation and expression within the arts modality [37,38,39]. In developmental disorders (autism, attachment trauma) patients often experience a disturbed sense of self and problems in the attunement with others. With their strong accent on shared improvisation practices, ATs offer a multitude of options to address issues, such as attunement in timing and use of space or attunement in emotional regulation [40,41]. Patients who suffered traumatic experiences may experience severe alienation from the social or cultural context. In these cases, ATs may address new ways into social and cultural involvement through shared art practices [42,43]. 

Within the set treatment goals and the patient’s capacities, the therapist will seek to address the patient’s potential for engaging in an aesthetic moment. Aesthetic engagement estranges or alleviates the patient from life as usual, which in the case of pathological stagnation can support patients to surpass familiar and automatized unhealthy action patterns [44]. Experts during FP and ES suggest that new authentic experiences occur throughout the explorations within the arts modalities and within the safe holding of the therapeutic alliance. In the enactive encounter the sensing while expressing body is locus of both action as well as conscious experience of the action [45]. These findings partly parallel Koch’s theoretical conceptualisation of working factors in ATs [46]. While dancing, singing, playing, expressing, the actions affect the participants’ perception, emotions and understanding within that situation [46]. The reflection on these processes by a patient individually and between patient and therapist may offer a hermeneutic understanding of the newly found potentials and evaluate their significance for therapeutic change [47]. 

### Towards a Tentative Conceptual Model

The specific contribution of the arts for the recovery of mental health and well-being may reside in the very fact that in the arts we live the simultaneous presence of somatic, emotional, social and cultural involvement. A vital aspect of all arts is that they offer the opportunity to articulate and express experiential content within the hermetic density of the embodied actuality [15]. It is (with)in this density that aesthetic choices emerge as evident and meaningful. The articulation and expression of lived experiences enter into the freshness of the aesthetic moment and the shared experiential space of the therapeutic dyad or group. The therapeutic context is composed such as to afford [48] specific experiential qualities for the participants to explore their capacities, to develop new action possibilities, to improve, to move and to move on. The arts modalities offer for the patient a potential space to develop towards self-actualisation and agency. 

From an aesthetic perspective, we may consider psychopathology as a person’s disconnectedness from a bodily felt sense of self. Arts therapists will guide participants to attentively follow their perceptions and bodily sensations. From these sensations participants can seek authentic forms of expressing in the arts modality and unfold the inner landscape into personal space and shared space. The disclosure of the experiencing self in the therapeutic space is then met by an aesthetically engaged therapist. Engaging the patient in animated and embodied experiences, the therapist supports the patient to connect to a corps vécu [49] and while playing and improvising with(in) the arts modality to (re)connect to a vital adaptivity in the present newness. This encounter can bring about a new, or newly connected, sense of self that is reflected in the person’s actions within the art modality. Arts-based activities offer the opportunity to (re)symbolise experiential content. Playing, dancing, painting, patients can practice the synthesis of experiences from life-history with their newly found explorations in the actual environment of the arts modality. In the interaction between patient and therapist these processes will be recreated over and over until the patient finds recovery or rebalanced mental health and can leave the therapy. 

ATs invite the patients’ potential to engage in aesthetic experiences. Once established, this engagement forms the base for the development of creativity and playful presence, cognitive engagement and reflexivity, decision making, regulation and performativity within the arts modality. Rather than treating pathological structures along psychological or medically informed procedures, ATs make an appeal on the healthy capacities of patients to engage in aesthetic immersion. This is what is considered here as the aesthetic turn to mental health. 

In the ATs, the therapeutic context is composed such as to afford specific experiential spaces for the patients’ personal development in accordance with the set therapy goals. In the creative encounter the therapist will guide the patients towards moments, that are close to their capacities to engage in aesthetic experiences but that hold at the same time a potential space, a zone of proximal development [50] into which they can develop their explorations within the arts modality. In the creative processes, they may find new possibilities to act and to symbolize or re-symbolise inner feelings or interpersonal relations. While dancing or playing they find and make (new) meaning from and within the experiential content. 

The alliance between patient and therapist is in ATs characterised by shared explorations and adaptive changes within the aesthetic moments. This is a unique therapeutic practice, as it is embodied and experiential by nature and exclusive to the co-created present moment [51]. There is a readiness to engage and act in the therapist as well as in the patient and an intersubjective engagement towards the context, that only will be revealed in the action within the aesthetic experience. This might be the intra-subjective perception and engagement of the individual during an action, like in solo improvisation or dance, or the intersubjective perception and engagement like in a group improvisation or group dance. From these experiences, the initially “unthought knowns” [52] (p. 277) can develop towards meaningful embodied knowns [53] in the experiential and reflective dialogue between patient and therapist. Implications of leadership styles or relational modes, like mirroring or witnessing only recently are taken into account in research on the effects of ATs [54,55]. The therapist’s approach to the therapeutic relation would make an interesting subject for future studies.

## 6. Concluding Remarks

### The Significance of an Aesthetic Framework for the Arts Therapies

Reflecting on arts-informed themes and procedures as they are described in the ATs literature and in ATs experts’ comments on meaningful interventions in ATs, this paper presented a tentative conceptual reframing of ATs contribution to the field of mental health in terms of aesthetics. The ATs offer a multitude of interventions which, when skilfully personalised, address in the patient lost or undeveloped capacities for multisensory or aesthetic engagement, for reconnecting with a bodily felt sense of self, for recovery of personal timing and personal space, for self-other relatedness and self-world relatedness, for creative elaboration of material and immaterial givens, for affirmation or rejection, for aesthetic insights [56]. All these aspects contribute to a healthy personality and their synergy leads to mental resilience and well-being [57]. 

An aesthetic perspective to mental health indicates a shift of paradigm towards positive mental health [58]. This shift parallels recent developments in the mental health field that emphasize supportive factors such as resilience rather than focussing on symptoms and pathology [59]. This goes along with a shift in diagnostic focus from pathological issues towards capacities, which matches well with the ATs perspective to support patients in their creative, expressive and performative capacities. The ATs seem well equipped to build therapeutic interventions from an aesthetic frame of reference. As has been shown, this aesthetic reference is not about beauty but it is in our perception of beauty or of meaningful aesthetics that we fully grasp the complex density of this matter in just one sigh, whereas the attempt to put this complexity into the linearity of words, still is a challenging endeavour. However, the conceptualisation and actual application of arts-informed and aesthetic procedures may be considered the distinctive contribution of ATs to the field of mental health. Further explorations of this topic may focus on the relevance of the presented conceptualisation for the therapeutic theoretical frame of reference for ATs and research on the efficacy of ATs interventions.

## Figures and Tables

**Table 1:** Art related activities and procedures in the arts therapies.

Activity/Procedure	In the Arts	In Arts Therapy
Invite	into space of potentials; to discover new possibilities	therapist as holding partner creates a situation that offers holding and opportunities for sensing & acting
Engage	into relation with materials, structures or partner	therapist creates the situation such as to afford development (into new action potentials)
Play	use an “as if” perspective	use of aesthetic illusion to alleviate from stagnation or illness
Explore	experiment with possibilities	experiment with new possibilities; try out and explore within enactive engagement
Replicate	repeat structures or motives that occurred to suit the artistic choices; remember and reproduce, building a repertoire	repeat meaningful patterns and structures that fit personal preference, the situation or combine interpersonal differences; cognitive engagement during remembering; develop enactive cognition and executive functioning; rehearse behavioural changes and develop mastery
Interact	address, show, connect, combine with partner’s materials	initially the therapist supports the patient to develop towards self-other-relatedness
Share	show, play together in duet/ensemble	performativity, self-other relatedness
Change	deliberately apply variations in structures, ways of playing/ways of moving	discover and explore alternative perspectives and actions
Improvise	while playing find new possibilities of expressing and articulating; connecting small meaningful entities	develop flexibility and flow of action; weaving threads from smaller entities; flow between interacting players
Select	select structures that fit the situation, or that are considered well suited to express personal experiences	learn to make decisions on what fit the personal preferences or capacities
Compose	combine findings from improvisations into meaningful entities, combinations	meaning making; agency to form a gestalt
Perform	show skills and expressive capacities	becoming visible, stand out
